# Supplementary material for: Historical Appreciation of World Health Organization’s Public Health Paper-34: Principles and Practice of Screening for Disease, by Max Wilson and Gunnar Jungner
Source: Int J Neonatal Screen. 2025 Jul 21;11(3):56. doi: 10.3390/ijns11030056 (PMC12286092; doi:10.3390/ijns11030056)
Supplement: Supplementary file 1 [file IJNS-11-00056-s001.zip › IJNS-3727330-supplementary.pdf]

1962 April/May

APPENDIX II

ITINERARY AND LIST OF PERSONS VISITED

Date	Place	Persons visited
April 2	WASHINGTON, D.C.	Daniel D. Swinney, Programme Director, Division of International Health
		Dr. James Watt, Chief, Division of International Health
		Dr. William H. Stewart, Chief, Division of Community Health Services Bureau of State Services
		Dr. Paul Q. Peterson, Chief, Division of Public Health Methods
April 3		Dr. Richard A. Prindle Deputy Chief, Division of Air Pollution Bureau of State Services
		Dr. Godfrey Hochbaum, Chief, Behavioural Sciences Section Division of Community Health Services
		Dr. Forrest E. Linder, Director, National Center for Health Statistics
April 4	BETHESDA, MARYLAND National Institute of Health:	Mr. William Haenszel, Chief, Biochemistry Branch National Cancer Institute
		Dr. Louis Thomas, Chief, Surgical Pathology Service Pathological Anatomy Department Clinical Center
		Dr. John C. Bailar, III Biometry Branch National Cancer Institute
		Dr. William J. Zukel Office of the Director National Heart Institute
		Dr. Harold Dorn, Chief, Biometrics Research Branch National Heart Institute
		Dr. Thomas D. Dublin, Chief, Epidemiology and Biometry Branch National Institute of Arthritis and Metabolic Diseases
April 6	NEW YORK, N.Y.	Office of Dr. Harald M. Graning Regional Health Director Public Health Service Room 900, 42 Broadway
		Dr. Arthur B. Robins Bureau of Tuberculosis Department of Health, New York City

Date	Place	No.	Persons visited
April 6 (contd.)	NEW YORK, N.Y. (contd.)	16	Dr. Oppenheim<br>Health Maintenance Clinics<br>Department of Health, New York City
		17	Dr. David Spain, Dept. of Pathology,<br>Beth El Hospital, Brooklyn
April 9		18	Dr. Milton Terris, Chief,<br>Chronic Diseases Unit<br>Public Health Research Institute<br>New York City
		19	Dr. Robert H. Greene and Dr. Mirick<br>Health Research Council<br>City of New York
April 10		20	Dr. Seymour Rinzler<br>Anti-Coronary Club<br>Kips Bay Health Centre, New York City
		21	Dr. Leopold Koss<br>Strang Cancer Prevention Clinic<br>New York Memorial Hospital
April 11		22	Dr. Menard M. Gertler<br>Department of Physical Medicine and Rehabilitation<br>College of Medicine<br>New York University
		23	Dr. Elaine Diacumakos<br>Sloan-Kettering Institute<br>New York City
		24	Dr. Louis Venet, Associate Director,<br>Strang Cancer Prevention Clinic<br>Memorial Hospital for Cancer and Allied Diseases, New York City
April 12		25	Dr. George Silver, Director,<br>Montefiore Medical Group<br>Montefiore Hospital, Bronx, N.Y.
		26	Dr. James McCarroll<br>Associate Professor,<br>Department of Preventive Medicine<br>Cornell University<br>Kips Bay Health Centre, New York City
April 13		27	Dr. Greenwald, Health Officer,<br>Tremont Health Centre, Bronx, N.Y.
April 16	4 FRAMINGHAM, MASS.	28	Dr. Ralph Paffenbarger, Chief,<br>Field Epidemiological Research Station<br>Public Health Service<br>Framingham Union Hospital
		29	Dr. William Kannel<br>Associate Medical Director<br>Heart Diseases Epidemiological Study<br>Framingham, Mass.
		30	Dr. Hugh Wilkerson, late, P.H.S.<br>Diabetes Epidemiology Study

April 17 5 **BOSTON, MASSACHUSETTS**

31 Dr. George Packer Berry, Dean,
Harvard Medical School
25 Shattuck Street

32 Dr. Arthur T. Hertig
Chief of Pathology
Harvard Medical School

33 Dr. Harry T. Phillips
Massachesetts State Health Department

Department of Preventive Medicine
Harvard University
34 Dr. Rutstein
35 Dr. Osler Peterson

36 Dr. Frederick J. Stare
Department of Nutrition
Harvard School of Public Health

37 Dr. Edward Kass
Mallory Institute of Pathology
Boston City Hospital

38 Dr. P. A. Yonge
Department of Obstetrics and
Gynecology, Harvard Medical School

April 19 6 **ALBANY, NEW YORK**

39 Dr. Franklyn B. Amos
Director of Professional Training
Health Education Service
84 Holland Avenue

40 Dr. Joseph T. Doyle
Cardiovascular Health Center
Albany Medical College

41 Dr. Robert Whalen, Medical Officer,
Albany County, N.Y.

42 Dr. Ward L. Oliver, Assistant Director,
Bureau of Adult Health and Geriatrics
New York State Dept. of Health

43 Dr. I. J. Brightman
Assistant Commissioner
Division of Chronic Diseases
New York State Dept. of Health

April 20 7 **WASHINGTON, D.C.**

44 Public Health Service:
Daniel D. Swinney, Programme Director

45 Dr. Leslie W. Knott, Chief,
Division of Chronic Diseases
Bureau of State Services
Room 3907 GSA Regional Office Building
7th and D Streets, S.W.

46 Dr. Robert L. Smith
Cancer Control Programme

47 Dr. Glen W. McDonald, Chief,
Diabetes and Arthritis Programme

48 Dr. Eugene Guthrie, Chief,
Neurological and Sensory Diseases
Service Programme

April 20 (contd.) WASHINGTON, D.C. (contd.)

49 Dr. David Brand
Heart Disease Control Programme

50 Dr. Claire Ryder, Chief, Services for
Long-Term Illness Programme

51 Dr. George Tokuhata, Special Assistant
to Division Chief for Technical
Development

52 Dr. Quentin Remein
Research Grants Officer

53 Dr. Wilfred David, Deputy Chief,
Chronic Diseases Division

54 Mr. Sauer, Statistics Branch
Heart Disease Control Programme

April 23

55 Dr. Alice Waterhouse, National Health
Survey, Public Health Service

April 26 7 PHILADELPHIA, PENNSYLVANIA

56 Dr. Katharine Boucot
and Dr. Laurence A. Browne
Women's Medical College
Henry Avenue and Abbottsford Road

57 Dr. Theodore H. Ingalls
Henry Phipps Institute
University of Pennsylvania
4219 Chester Avenue

58 Dr. William Weiss
Pulmonary Neoplasm Research Project

59 Dr. Don A. Mills
Community Health Services
Philadelphia Department of
Public Health
500 South Broad Street

April 27

60 Dr. R. R. Rascoe
104 Dulles
Hospital of the University of
Pennsylvania
36th and Spruce Street

61 Dr. Thomas Clarke
Diagnostic Clinic
Hospital of the University of
Pennsylvania
36th and Spruce Street

62 Dr. Ingram, Deputy
Health Commissioner,
Philadelphia Department of
Public Health
502 City Hall Annex

63 Dr. Irena Koprowska
Department of Cyto-Pathology
Hahnemann Hospital

April 30 8 BUFFALO, NEW YORK

64 Dr. James A. Dolce
First Deputy Commissioner
Erie County Health Department
601 City Hall

April 30 (contd.) BUFFALO, NEW YORK

65 Dr. Warren Winkelstein, University of Buffalo Chronic Diseases Institute

66 Dr. Morton Levin, Kress Institute
Roswell Park Hospital

67 Dr. Ruth and Dr. Harry Graham
Roswell Park Hospital

68 Dr. Krauss, Medical Director,
Well-Ageing Clinic
Erie County Health Department

May 2 9 ANN ARBOR, MICHIGAN

69 Dr. Myron E. Wegman, Dean,
School of Public Health
University of Michigan
109 South Observatory Street

70 Dr. Frank Reynolds
School of Public Health
University of Michigan

May 3

71 Dr. G. Hoyt Whipple
School of Public Health
University of Michigan

72 Dr. John Tupper
University of Michigan Staff Health Scheme

73 Dr. Frederick Epstein
Tecumseh Project
School of Public Health

74 Dr. Virgil Slee
Professional Activities Study
Ann Arbor

75 Dr. Millicent Payne, Tecumseh Project

May 4

76 Dr. Vlado Getting
Professor of Public Health Practice
School of Public Health

77 Dr. Dodge, Tecumseh Project

78 Dr. A. Donabedian
Bureau of Public Health Economics
School of Public Health

May 7 10 CHICAGO, ILLINOIS

79 Dr. Arthur B. Price
Regional Health Director
Public Health Service

80 Dr. Oglesby Paul
Western Electric Cardiovascular Survey

May 8

81 Dr. Jeremiah Stamler
Heart Disease Control Programme
Chicago Board of Health

82 Dr. Elizabeth McGrew
Department of Pathology
University of Illinois College of Medicine

May 8 (contd.) — CHICAGO, ILLINOIS (contd.)

83 Dr. Saphir
Department of Pathology
Michael Reese Hospital

May 9

84 Dr. George Wied
Department of Pathology
Chicago Lying-In Hospital

May 10 — BERKELEY, CALIFORNIA

85 Dr. B. K. Milmore, Chief,
Bureau of Chronic Diseases
California State Health Department

86 Dr. John Goldsmith, Chief,
Section of Air Pollution
State Health Department

87 Dr. J. E. Dunn, Junr., Chief,
Section of Cancer Control
State Health Department

May 11

88 Dr. Yerushalmy
Department of Epidemiology
University of California School of Public Health

89 Dr. Stallones
Department of Epidemiology
University of California School of Public Health

90 Dr. Finan, Chief,
Human Population Laboratory
Division of Preventive Medical Services
California State Health Department

May 14

91 Dr. Lester Breslow, Chief,
Division of Preventive Medical Services
California State Health Department

92 Mr. Jack Kirkpatrick, Administrator,
Division of Preventive Medical Services
California State Health Department

May 14 — OAKLAND, CALIFORNIA

93 Dr. Morris F. Collen
Permanente Medical Group
Kaiser Foundation Health Plan

May 15 — SAN FRANCISCO

94 Dr. David Wood, Chief,
Cancer Research Institute
University of California Medical Centre

BERKELEY

95 Dr. Borhani
Heart Disease Control Section
California State Health Department

96 Mr. Jack Nelson
Blindness Prevention Programme
California State Health Department

May 16		SAN FRANCISCO	97	Dr. Seymour M. Farber<br>Professor of Internal Medicine<br>University of California<br>County Hospital
			98	Mr. Samuel Pharr, Senior Technician,<br>Bacteriology Laboratory<br>County Hospital
			99	Dr. Roger Wilson<br>Department of Epidemiology<br>University of California Medical Centre
			100	Dr. Eileen King<br>Department of Pathology<br>University of California Medical Centre
May 17	14	STANFORD UNIVERSITY MEDICAL SCHOOL, PALO ALTO	101	Dr. Charles E. McLennan<br>Professor of Obstetrics<br>Stanford University Medical School
	15	SAN JOSE	102	Dr. Miller<br>San Jose City Health Department
May 18		SAN FRANCISCO	103	Dr. Ray H. Rosenman<br>Harold Brunn Institute<br>Mt. Zion Hospital
[illegible]ay 21	16	SANTA MONICA	104	Mrs. Edith Pross<br>Los Angeles City Health Department -<br>Santa Monica General Hospital Continuity of Nursing Project
	17	LOS ANGELES	105	Dr. Edward Gomer Jones<br>Professor of Gynaecology<br>Los Angeles County Hospital
May 22			106	Dr. L. Goerke, Chairman,<br>Department of Preventive Medicine and Public Health<br>University of California at Los Angeles
			107	Dr. J. M. Chapman<br>Department of Preventive Medicine and Public Health<br>University of California at Los Angeles
May 23	18	SAN DIEGO	108	Dr. P. L. Martin and Dr. T. A. Slate<br>Gynob Laboratory
May 24	19	LA JOLLA	109	Dr. Sheldon C. Sommers<br>Department of Pathology<br>Scripps Memorial Hospital
May 26 - 28	20	VANCOUVER	110	Dr. D. A. Boyes<br>Cancer Research Institute of British Columbia
			111	Dr. H. K. Fidler<br>Department of Pathology<br>Vancouver General Hospital
